# Participation in healthcare consultations: A qualitative study from the perspectives of persons diagnosed with hand osteoarthritis

**DOI:** 10.1111/hex.13744

**Published:** 2023-03-14

**Authors:** Hege Johanne Magnussen, Ingvild Kjeken, Irma Pinxsterhuis, Trine Amalie Sjøvold, Toril Hennig, Eva Thorsen, Marte Feiring

**Affiliations:** ^1^ Department of Rehabilitation Science and Health Technology, Faculty of Health Sciences Oslo Metropolitan University Oslo Norway; ^2^ Norwegian National Advisory Unit on Rehabilitation in Rheumatology Diakonhjemmet Hospital Oslo Norway; ^3^ Norwegian National Advisory Unit on Rehabilitation in Rheumatology REMEDY, Center for treatment of Rheumatic and Musculoskeletal Diseases, Diakonhjemmet Hospital Oslo Norway; ^4^ Martina Hansens Hospital Sandvika Norway; ^5^ Lillehammer Rheumatism Hospital Lillehammer Norway

**Keywords:** agenda‐setting, chronic condition, decision‐making, hand osteoarthritis, healthcare consultations, self‐management

## Abstract

**Introduction:**

Scarce health resources and differing views between persons with hand osteoarthritis (OA) and health professionals concerning care preferences contribute to sustaining a gap between actual needs and existing clinical guidelines for hand OA. The aim of this study is to explore the experiences of persons diagnosed with hand OA in their encounters with health services and how those experiences influence negotiations and decision‐making in hand OA care.

**Methods:**

Data from 21 qualitative interviews with persons diagnosed with hand OA were collected, transcribed verbatim and analysed using reflexive thematic analysis.

**Results:**

Three main themes were developed: symptoms are perceived as ordinary ageing in everyday life, consultations are shaped by trust in healthcare and the responsibilities of prioritisation and self‐care govern interactions.

**Conclusion:**

Ideas of ageing, professional knowledge and self‐management dominate hand OA health encounters and contribute to shaping illness perceptions, preferences and opportunities to negotiate decisions in consultations.

**Patient or Public Contribution:**

Two patient research partners with hand OA are members of the study project group. One of them is also a co‐author of this manuscript.

## 1 INTRODUCTION

The global burden of osteoarthritis (OA) accelerates with an ageing population,^1^ posing challenges for health services.^2^ Hand OA services aim to care, not cure. Progress is limited in developing effective treatments.^3^, ^4^ While hand OA has recently gained increased attention,^5^ interventions do not fully consider persons with hand ailment in their encounters with the health system.^6^ Poor access to treatment,^5^ low consultation rates,^7^, ^8^ delayed contact with health services^9^, ^10^ and symptoms perceived as inevitable with ageing^11^ are reported. This contributes to hesitance in seeking health services attention^12^, ^13^ and beliefs that the condition does not justify treatment.^7^, ^14^

The health‐seeking process is complex,^15^ and people with hand symptoms report a lack of information^16^, ^17^ and dissatisfaction with consultations and treatments,^18^, ^19^ contributing to their experiences of having a condition that does not get the attention it deserves.^9^ Differences between people with hand symptoms and health providers on OA care^16^, ^20^, ^21^ underline the importance of enhancing the perspectives of people with hand ailment to reduce the gap between their perceived needs and existing guidelines for hand OA. Current research is limited to experience with recommended treatments, where shared decision‐making^22^ does not fully address the complex nature of negotiations and governing motives in consultations. Our study aimed to contribute to the limited set of studies on decision‐making in healthcare consultations from the perspective of persons with a chronic condition.

We saw consultations between people with hand ailments and health professionals as encounters where negotiation takes place. Erving Goffman^23^ inspired our understanding of the concept of ‘encounter’ as a social organisation where certain rules exist. Thus, consultations encompass obligations and expectations, serving as a location for interaction.^23^ By using the concept of encounter, we highlighted consultations as structured and governed by layers of rules, norms and societal values while also acknowledging that negotiations within encounters are less formal and point to adjustments made in consultations to comply with institutional demands.^24^

We included power dimensions to our analysis through Steven Lukes'^25^ work to enable situations of ‘being liberated from certain power relations and of the reduction of power within a relation’.^26^,p.27 We argue that hand‐OA discursive ideas influence participation. Lukes' emphasis on power dimensions is useful to our analysis as it allows us to think through how power is exercised within healthcare, where asymmetry and power in relations might be difficult to observe. This conceptualisation of power is generative for grasping how power relations play out, impact encounters and subsequently influence negotiations to reach decisions in healthcare.

Lukes'^25^ three power dimensions are decision‐making, agenda setting and ideological power. The decision‐making power, overt and direct, aims to reach specific results in conflict situations. The agenda‐setting power, indirectly exercised, portrays how decisions linked to the potential conflict are prohibited from being taken. Ideological power, concealed and embedded in structures and institutions, rises beyond individual actors so that the exercise of power is taken for granted.

The decision‐making process in healthcare has gradually shifted from one of the passive patients to one of the responsible patients,^27^ with the aim of redistributing power.^28^, ^29^ These processes, framed within the democratisation of health services, contribute to new understandings of health encounters.^30^ By linking actual negotiation processes in encounters with larger structural considerations of power, we ask how decisions are made in hand OA healthcare consultations.

## 2 METHODS

### 2.1 Context

The Norwegian state plays a central role in providing access to fundamental goods, including healthcare.^30^ In Norway, due to poor access to relevant hand OA services in primary healthcare, persons with hand symptoms are referred for hospital consultations with rheumatologists and occupational therapists. The two hospitals from which participants in this study were recruited specialise in rheumatology and were chosen based on their similarities in providing services to persons with hand OA while also featuring different local processes.^31^ The diagnosis is made based on patient history and clinical examination.^32^ Symptoms are hand pain and stiffness, which affect one in two women and one in four men.^33^ Recommended general treatment includes information on hand OA and exercises for the hands, whereas orthotics, pain medication and surgery are considered individually.^5^

### 2.2 Research team

This study is part of a three‐phase project that aims to understand current hand OA pathways, including patient experiences and professional practices. A randomised controlled trial (RCT) was initiated in 2017 (400 participants),^34^ followed by an ongoing qualitative study and a Delphi consensus process planned from 2023. The first author, H. J. M., is a PhD student and a physiotherapist with 20 years of clinical and managerial health and humanitarian experience. The co‐authors include professors, an associate professor, clinicians and a patient research partner. We also consulted with an international advisory board of researchers with various professional backgrounds.

### 2.3 Participant recruitment, eligibility and demographics

Between December 2020 and December 2021, we recruited 21 persons diagnosed with hand OA who had received primary and specialised healthcare. An occupational therapist in each hospital identified participants and informed them about the study before their inclusion. These gatekeepers were instructed to draw on varying age, gender and hand OA duration in the purposive participant sampling. Twelve participants were recruited from one hospital based on prior inclusion in the completed RCT. The other nine participants were recruited from a hospital in a different geographical area based on their participation in a hand OA education programme. Interviews were scheduled within 1 month from recruitment. Two persons withdrew prior to interviews, reporting time constraints and long‐term illness. To reduce the risks of obligatory participation when recruited by an occupational therapist on whom they depended for services, H. J. M. presented study objectives, consent form and her nonaffiliation with the hospitals to participants before interviews. Fifteen women and 6 men aged 47–86 were included. They had symptoms that had been present for the previous 2–20 years. Participants included 13 in retirement, 5 in full‐time positions, 1 jobseeker and 2 with disability benefits. Only two participants were under 60 years, which may be due to perceptions linking symptoms to ageing and subsequent delays in seeking healthcare. Additionally, we only succeeded in recruiting one participant with an immigrant background, which may reflect barriers to accessing specialised healthcare and participating in research for immigrants. Busy clinical gatekeepers may also have resulted in recruiting those most accessible.

### 2.4 Data collection

Through a qualitative research design, informed by a constructionist epistemology,^35^ we collected data using qualitative interviews. An interview guide (Supporting Information: Appendix 1), piloted with two patient research partners, including content about initial contact with health services, encounters with health professionals, treatment and self‐care strategies. Nineteen interviews took place in person, while two participants chose digital interviews. One participant had his spouse present upon request. Interviews lasted 55–90 min each and were audio‐recorded. Data were stored on the secure platform services for sensitive data, in compliance with the Norwegian privacy regulation, including immediate encrypted audio files transfer post‐interviews. Participant anonymity was safeguarded through separate participant information and data file storage. Through research team discussions, information power was reached after 21 interviews.^36^ The broad study aim and cross‐case analysis required more participants, while H. J. M.'s experiences as a qualitative researcher with some theoretical knowledge and skills to establish a good dialogue called for fewer participants.

### 2.5 Data analysis

We applied reflexive thematic analysis^37^ to endorse the process of researcher subjectivity and the situated generation of knowledge to report patterns.^38^ NVivo (released in March 2020) was used to structure the data. H. J. M. conducted all interviews and subsequent verbatim transcriptions,^39^ reading and rereading transcripts to become familiar with the breadth and depth of the content while taking reflective notes. Postinterview debriefs with co‐authors took place to emphasise how H. J. M. influenced the research process and data construction. Our orientation to data was to interpret meaning beyond what participants explicitly communicated. We engaged empirical data and theoretical understandings in parallel.^40^ H. J. M. developed semantic and latent codes inductively from reading the data, alternating with personal experiences and academic literature with a focus on microlevel interactions.^41^ After several rounds of research team discussions, including one session discussing two different interview transcripts in detail, we sorted main codes into potential themes before presenting preliminary results to the advisory board. Themes were thereafter reviewed and refined to ensure relevance to the coded extracts and that we had captured patterns of shared meaning across the data set.^42^ In reviewing themes, we added power dimensions to our analysis in an effort to grasp the complex decision‐making process where sociocultural factors were seen to influence perceptions and actions. This iterative process helped us to gain a deeper understanding of the themes and how they connect in telling an overall interpretive story (Table 1).^43^

**Table 1:** Codes and themes development.

Examples of data‐driven recurring codes	Refined codes	Preliminary themes	Themes
Hand OA is a common condition	Adapting to the ailment	The common person with a hand ailment	Symptoms of ordinary ageing in everyday life
Changes in hands go unnoticed	Does not regard own ailments as illness		
Have gotten used to the discomfort	Minimising the severity of the condition		
Hand OA is not a severe condition			
Found ways to live with hand OA			
Hand ailment addressed by chance	Serendipitous ways to health attention	The fortunate person with a hand ailment	
Hand ailment expected with age	Does not deserve healthcare	The unobtrusive person with a hand ailment	
Do rarely make use of health services	Having a disease with low status		
	Delay in initiating contact with health services		
Challenging to speak up about hand ailment	Moderation in health encounters		
Difficult to know what questions to ask in consultations			
Discomfort difficult to explain			
Could have made stronger demands in consultations	Does not want to become a liability		
Don't want to be seen as a difficult patient			
Open‐minded towards health services upon entering	Placing confidence in health service	The trusting person with a hand ailment	Consultations shaped by trust in healthcare
Confidence in people who know what they do	Health professionals have expert knowledge		
In the hands of competent professionals	Confidence in healthcare professionals to safeguard their interests		
Respecting the work of health professionals			
Entered the consultation with a blank slate	Underestimating knowledge about one's own illness		
Agreeing with what health professionals recommend	Health professional agenda setting		
	Healthcare providers are gatekeepers to goods and services		
	Dependence on health professionals		
Health professionals showed an interest	Well‐being in consultations with health personnel		
Importance of being believed in consultations	Health professional's recognition of hand ailment		
Health professionals provided explanations	Recommendations from healthcare professionals are taken seriously		
Health professionals came up with solutions	Health professionals are seen to safeguard patient interests		
Do not want to be a burden	The ideal is to be a considerate patient	The responsible person with a hand ailment	The responsibilities of prioritisation and self‐care govern interactions
Politeness in consultations	Comparing one's own needs with the needs of others		
Others with larger needs deserve healthcare more	Do not want to burden the healthcare system		
Other own ailments are more in focus	Priorities amongst own conditions/ailments		
Few opportunities to get better	The initiative is with the person with a hand ailment		
No available treatment	Responsibility for providing relevant information in consultations		
Own effort expected	Lack of own openness reason for not receiving relevant support		
Home exercises are the only solution			
Active in consultations			
Hand OA is one's own fault	Own fault that the illness progressed		
Could have spoken up earlier	Delay in health seeking		
One must ask to get answers	Lack of preparedness in advance of consultations		
Not well prepared for consultations	Own responsibility when improvement is not achieved		
Not good enough to follow up on recommendations			
Keep consultation time	Accountable to healthcare professionals		
Health professionals have a busy schedule	Do not want to burden the health system		
	Facilitates efficiency at the expense of one's own needs		

Abbreviation: OA, osteoarthritis.

## 3 RESULTS

**Figure 1:**
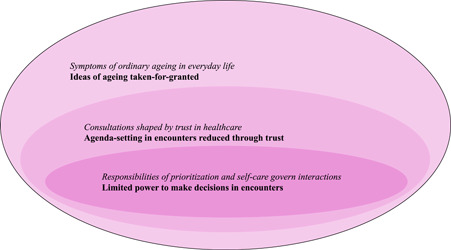
Illustration of the study results, inspired by Goffman's encounter concept combined with Lukes' power dimensions.

We developed three main themes from codes as presented in Table 1 to capture how decisions are made in healthcare consultations (Figure 1). First, persons with hand ailment bring taken‐for‐granted ideas about *symptoms of ordinary ageing in everyday life* (outer circle) into *consultations that are shaped by trust in healthcare* (middle circle). This limits the power to make decisions in encounters when the *responsibilities of prioritisation and self‐care govern interactions* (inner circle).

### 3.1 Symptoms of ordinary ageing in everyday life

Hand pain and functional limitations were described by many participants as common, a product of ageing and nontreatable, contributing to limit contact with health services: ‘There is nothing that can be done about it, and it is so common. And of course, when you age as well, then there is more of it’ (Woman86). When linking it both to ageing and the notion that nothing can be done to address the challenges, she reduces the hand OA significance and accompanying needs. This might explain why most study participants also described symptom onset years before initiating contact with the healthcare system, keeping diffuse symptoms to themselves.

Some participants frequently used the word discomfort rather than pain in interviews when they talked about their hand condition, further underplaying the severity. ‘Well, it probably has something to do with age and the fact that I am more than 75 years old and have experienced almost everything, for better or worse. So, that is how it is, and if you don't have more severe pain than what I have, then I must live with that’ (Man75). The man also points to old age as a factor in explaining hand symptoms. Simultaneously, he highlights a situation that he takes for granted and adapts to without expecting health interventions.

When participants finally entered health services, it was often in conjunction with another health‐related matter, substantial symptoms of deterioration or by chance. As one participant with an accident resulting in a thumb fracture for which X‐rays identified the hand OA said: ‘I must feel like I am ill to go to see a doctor. I have probably not perceived this as being illness and that might be one of the reasons for not following up on it myself or making demands in consultations’ (Man70). This man had noticed changes in his hands for years prior to the accident and had not considered investigating them further or positioning the condition of his hands within an illness narrative. Another participant said the following: ‘Even though my hands hurt, I am not ill’ (Woman68). When discomfort does not constitute illness, it becomes difficult to justify seeking healthcare, strengthening the taken‐for‐granted notion of not being entitled to healthcare. This presents challenges for having the condition assessed and managed on time.

### 3.2 Consultations shaped by trust in healthcare

Participants in the study talked about trust on various levels, from their trust in the overall health system through trust in health providers as a professional group to trusting individual health providers. As one participant said: ‘I do have this genuine trust in the healthcare system telling me that if I can make it inside, I will be well cared for’ (Man70). This man channels the genuine trust embedded in him to the system level of healthcare, expressing confidence and good faith as part of health‐seeking, expecting to be cared for upon entering. Through this trust, he attributes good intentions to the healthcare system for assisting him with hand changes that he is no longer able to fully understand or manage. Correspondingly, another participant said that he was not in the driver's seat, further illustrating how participants leave it to health professionals to set the agenda for consultations. This social form of trust provides scaffolding for interactions and was also expressed by one participant who, at the same time, extended her trust beyond the institution.

In general, I have trust in the healthcare system and in the people who are there because they know their jobs. I believe that trust is important to bring along. I am not very sceptical, like why are they saying that, why are they doing this. People talk and are present and provide information, and that brings trust, I think. In addition, I know that they are professionals. (Woman73)

This woman extends her trust in the institutional health system to the individuals who conduct the work. Her trust in their expertise is based on their knowledge and skills, and she highlights their positions as professionals as a factor in trusting them. Participants in the study considered health professionals as experts in addressing hand OA challenges.

However, several participants said that they were not comfortable expressing their own opinions beyond politeness during consultations and, in retrospect, could have made stronger demands. They found it difficult to know what questions to ask when marginalising their own knowledge about hand OA, as conveyed by one participant: ‘I have not been in what we can call situations of conflict with health professionals in that way. Absolutely not. It has been like an open dialogue. For the most part, I have agreed to what they have suggested. I don't have the knowledge to oppose that’ (Man70). He talks about an open dialogue where consensus prevails and where he does not question professional suggestions in consultations due to his own lack of knowledge. Thus, professional knowledge is not up for debate when taken for granted by the participants.

The trust described by participants is present independently of the health system level or profession and further encompasses personal trust in individual health providers: ‘She is genuinely nice like that, sees you and is present. Yes. Like it is just you there. Not all the others, like it is just you’ (Woman68). This participant points to the health providers' ability to acknowledge the symptoms and to recognise the participant as a unique individual. In this way, the participant experiences being the focus of the consultation through the professional and interpersonal skills of the health professional.

Some participants also referred to situations in which trust was absent. The two participants on the state disability pension, for example, expressed a lack of trust in the health system stemming from not being trusted by that same system. They do not trust an institution they believe questions their narrative, while at the same time expressing positive experiences with health professionals in hand OA consultations: ‘It was good for me that someone said, “I see that it is painful.” I felt I got help, really. And was believed in. That is the most important thing. To be believed in’ (Woman60). In this consultation, the lack of institutional trust was outweighed by trust in an individual health provider with the ability to acknowledge the woman's pain, demonstrating how trust in an individual can be present despite the absence of institutional trust.

### 3.3 Responsibilities of prioritisation and self‐care govern interactions

Notwithstanding the trust, participants also described a social responsibility when comparing their own illness with others whom they perceived to need healthcare more: ‘I think it is something inside me telling me that I should not annoy that doctor. Because he probably has many others who are more ill than I. That is where I am at, yes’ (Woman70). This illustrates how the participants' social responsibility to not burden the healthcare system is prominent when pointing out that other individuals deserve medical attention more than she does. Thus, participants see it as an obligation on behalf of society at large to downgrade their own needs by prioritising. This loyalty towards society shows a complex process of seeking and receiving healthcare, where their understandings and actions cannot be detached from the environment in which their lives play out. Consequently, moderation in seeking and obtaining healthcare prevails. Moderation in encounters is accentuated amongst numerous participants beyond the downgrading of their own condition in comparison with others, as shared by another participant:

Well, there is this thing about being open about my situation. I know that myself. I am not open about myself all the time. That I can exit the doctor's office and think to myself, why did I not address that? But it is something about the time they have set aside. You know that they have scheduled a fixed time. And then you are not supposed to exceed their time. (Woman72)

This woman finds it challenging to bring her hands to the health professionals' attention. She remains quiet about her own needs in an effort to avoid becoming a burden. Upon exiting the consultation, she reflects on why she did not bring forward concerns about her hands and pointed to a time. In this way, time becomes a responsibility factor when participants talk about the importance of not exceeding consultation time. They express an obligation to keep consultations short, adhering to the rules of the encounter. Hence, the norm of time outweighs one's own concerns and becomes a barrier to present needs.

In contrast, one younger participant in the study who found it challenging to conduct his work as a craftsman said: ‘I can sit for a long time if there is a need for that. Others must wait, then. When it is my turn’ (Man58). The man is not concerned with consultation time and does not adapt his own needs to the health provider's schedule. As such, the responsibility to address one's own needs triumphs over the health providers' busy schedule and societal needs. One explanation for his differing opinion might be that there is more at stake for people for whom functional hands are a prerequisite for employment and income. Accordingly, a moderate approach is replaced by more direct attention to one's own situation when decisions are made in consultations.

With few treatment options available for hand OA, most participants talked about a responsibility to respond to their own needs, as expressed by one participant: ‘You should preferably get well by yourself’ (Man71). The man points to self‐care as the proper way to respond to needs in the absence of other relevant treatment. In this way, few expectations exist for health system interventions. This social responsibility to prevent social expenses on healthcare is seen as directing personal decision‐making processes. By taking on individual responsibilities for self‐care, participants at the same time attribute a lack of progress and result in managing the condition to their own lack of initiative.

## 4 DISCUSSION

Our study aim was to explore the experiences of persons diagnosed with hand OA in their encounters with health services and how these experiences influence decision‐making in hand OA care. The results show that people with hand ailments bring trust into encounters with health professionals for a condition they perceive to be age‐related and ordinary. They also give accounts of responsibilities to prioritise and self‐care in a process shaped by hand OA as a chronic condition, influencing the definition of needs, how those needs are responded to and by whom.

### 4.1 Ideas of ageing taken for granted

In this study, participants viewed symptoms as part of ordinary ageing. Moreover, they downplayed severity, which strengthened the notion of not being entitled to healthcare when comparing their own situation with that of others. This aligns with other studies,^15^, ^44^ in which the perceived worthiness of the illness was judged through social comparison when considering whether to consult health services. The ‘ordinary ageing’ narrative generated in our analysis corroborates previous findings.^11^, ^12^, ^45^ The meaning our participants attributed to symptoms is an ongoing and complex social process. It is influenced by how symptoms are perceived within a wider socio‐political context where power over worldview, as Lukes^25^ writes, contributes to shaping the roles and identities of ageing people with chronic conditions.^46^

Participants similarly pointed to the ordinary role that hands play in everyday life despite symptoms, referring to diffuse and unnoticed changes. When considerations of healthcare attention are marginalised, and ageing takes prominence, the actions of persons with hand ailments are shaped prior to, during and after consultations. As such, they accept their position within an existing order and bring society's view of themselves into encounters, as said by Goffman.^23^ When wider society's identity beliefs position hand OA within a natural ageing frame, symptoms are accepted as normal. Contrasting Henselmans and colleagues'^47^ reports of active patients with chronic illnesses in consultations, our analysis shows how moderation in the illness experience impacts the actions taken, if any, by persons diagnosed with hand OA.

Severity and acuteness dominate health policy priority‐setting and resource allocation.^48^, ^49^ Our participants conveyed that other people with more severe conditions should have priority over them and deserve healthcare more. In this way, participants in our study acted on the limited power given to them by overarching health priorities, positioning hand OA at the lower end of the prestige hierarchy^50^ and thus surrendering their spot to others becomes the action. As such, participants get responsibilities beyond catering to their own needs when they feel obliged to also preserve collective interests. Our results show attitudes of modesty as a response to efforts to align with expectations.

When a hegemony linking hand OA with ageing and the low societal priority becomes significant in regulating how persons diagnosed with hand OA negotiate in consultations, the characteristics and stability of such encounters are intertwined with the wider social world. We argue that accepted understandings in society regarding ageing govern interaction in consultations.

### 4.2 Agenda‐setting in encounters reduced through trust

In our study, trust in health professionals as experts dominated encounters. Grimen^26^ argues that there are few alternatives to trusting in interactions between patients and health professionals, where patients are structurally inferior and dependent. He points to a knowledge gap, making it difficult for patients to challenge health professionals' judgements. As such, the taken‐for‐granted position of epistemic superiority of health professionals over patients shapes the definition of and response to needs.^51^

The literature points to an understanding of health professionals as experts,^15^, ^52^ where high trust levels coincide with a passive patient role^53^ and where unvoiced patient agendas in consultations influence outcome,^21^, ^54^ resonating with our study results where health professionals control the agenda. As such, the dominant values and beliefs embedded in expert knowledge shape the consultation. The domination of the task‐oriented agenda of health professionals based on technical skills and clinical guidelines oriented towards the disease is reported in several studies^20^, ^55^ and might not cohere with the agenda of the silent person with an illness.^21^

Conversely, Porcheret and colleagues^56^ found shared preferences for a biomedical approach during OA consultations, while Feddersen and colleagues^57^ found that the biomedical knowledge of nurses facilitated dialogue on the illness experience of women with rheumatoid arthritis, resulting in shared decision‐making. Our results, in contrast, show a knowledge gap in which persons diagnosed with hand OA talk about their own lay knowledge as substandard compared with health professionals' elevated knowledge.

Additionally, when hand OA consultations become procedural, the negotiation space of patients narrows as adherence to rules defines action more than active and negotiated processes. This leaves aspects unspoken in consultations, consequently preventing decisions from being made when applying Lukes'^25^ agenda‐setting power lens to the participants in our study saying that they could have made stronger demands in consultations in which they did not actively control the time or direction. This shows how dynamics in encounters are framed by invisible structures governing the actors' thoughts and actions to sustain order.

The perceived lay knowledge inferiority might also contribute to the consensus portrayed by participants when health professionals' opinions are taken for granted, which is harmonious with Lukes'^25^ third dimension of power. As such, an absence of conflict prevails in interactions, even though the interests of persons diagnosed with hand OA might not be in line with health professionals' knowledge or actions. This resonates with a study by Lian and colleagues^58^ in which patients in consultations responded politely to questions, rarely asking questions and making few attempts to set the agenda. In our study, when persons diagnosed with hand OA did not express their concerns, those concerns were not addressed.

Although trust within healthcare is seen as contributing to health system functioning^59^ and enhanced health outcomes,^60^ we argue that trust also contributes to sustaining the agenda‐setting power of health professionals when persons with hand ailments take expert knowledge for granted. Expert knowledge reinforces the power hierarchy when persons diagnosed with hand OA influence healthcare provisions less than the health professionals with whom they interact.

### 4.3 Limited power to make decisions in encounters

Our study shows how participants describe managing hand OA on their own when few other options are made available to them. In this process, they also become accountable for the lack of improvements when the aim is to care for, not cure, hand OA. Support for self‐management is recognised as central in responding to chronic health needs.^61^ Self‐management in hand OA care aims to improve patient autonomy,^5^ and shared responsibilities between health professionals and patients are reportedly facilitating self‐management in rheumatology.^62^ Subsequently, the decision to self‐manage hand OA can be viewed as a shared responsibility where negotiation in consultations evolves around the interests of persons with hand OA.

Concurrently, significant disparities have been reported between patients with arthritis and health professionals regarding whether support for self‐management has occurred.^63^ Moreover, what happens in consultations is influenced beyond individual interactions through socio‐political factors. Norwegian health policy and practice position self‐management as central in responding to growing demands for healthcare even though there is inconclusive evidence to support the cost‐effectiveness of such approaches.^61^, ^64^, ^65^ Self‐management strategies can be seen as shifting responsibilities from policy and professional levels to individuals,^66^ in line with the experiences of participants in our study. Thus, the allocation of resources becomes a driving force more than the actual needs of individuals with chronic conditions, contributing praise for those who have the capacity to take on such responsibility while marginalising those who do not.^67^, ^68^

Persons with ordinary diseases that are expected with age and have no cure in our case accepted self‐management within a frame of patient autonomy and the politics of health resources. In this way, the interests of persons with chronic diseases are shaped by pre‐existing and overarching ideological patterns in society, presenting self‐management as the norm in chronic care. Thus, the interests of persons diagnosed with hand OA are silenced by larger societal considerations where a transfer of responsibilities in the name of patient autonomy and empowerment can be seen as influencing persons with a chronic condition to accept self‐management.

Even though the intentional actions of persons with a chronic hand condition cannot be excluded from consultations, we argue that negotiations in hand OA care, when exercised under dominant age and self‐management influences, limit the agenda and participation of persons with perceived age‐related chronic conditions in defining and responding to needs.

### 4.4 Strengths and limitations

The present study addresses a knowledge gap by shedding light on multiple factors influencing consultation dynamics and opportunities for decision‐making in hand OA care, which might be relevant given the large population of persons with chronic conditions. While a single analyst allowed for prolonged and deep engagement with the data, continuous discussions between authors throughout the analytical process generated important reflections about H. J. M.'s engagement with participants and the data.

Although Goffman^23^ and Lukes^25^ provided the lens for understanding interactions in consultations, certain facets of the results were not captured through encounters or power dimensions. The lack of trust by participants in disability pensions and the experiences of individual needs outweighing social responsibilities are examples of how participants also break norms and make independent choices when they act on unstable and varying interests influenced by relations and circumstances.

We recognise that the time between symptom onset and consultations, as well as experiences with other conditions and services, shaped what our participants found acceptable, emphasised and conveyed during interviews. This might be a limitation, but it might also strengthen potential applicability to chronic conditions beyond hand OA.

## 5 CONCLUSION

Our study shows how symptoms are seen as ordinary and expected with age, which subsequently delays health‐seeking and influences decision‐making. The trusting person with a chronic hand condition rarely sets the agenda in encounters with health professionals when negotiating over a condition expected with age and with few interventions beyond self‐management. As such, persons with chronic conditions become responsible for addressing their own needs. We highlight health consultation complexities with relevance for persons with chronic conditions, health professionals and policymakers when optimising clinical practice and active participation, contributing to reduce gaps between patient needs and clinical recommendations. Stronger awareness amongst health professionals about power dimensions in consultations can accelerate opportunities for persons with chronic conditions to increasingly influence the consultation agenda and outcome. Moreover, alternatives to prominent self‐management approaches, such as increased health professional involvement for patients needing stronger support, should be considered in providing relevant and equitable healthcare.

## CONFLICT OF INTEREST STATEMENT

The authors declare no conflict of interest.

## ETHICS STATEMENT

The Regional Committee for Medical and Health Research Ethics (case number 2017/742, 2020/8450) and the Norwegian Centre for Research Data (reference number 197320) approved the research project, which was conducted according to the principles of the Declaration of Helsinki. Written informed consent was provided by participants before interviews and continuously negotiated. We informed participants of their rights to withdraw from the study anytime, without consequences, and that their data would be anonymised. We strived to be well prepared, attentive to participants, maintain their integrity and continuously reflect on the processual, relational and situational nature of the study. For example, when participants expanded their illness experiences well beyond hand osteoarthritis, H. J. M. made efforts to respectfully get back on track. Even though H. J. M. is also a health professional, the researcher's role was sustained throughout the interviews. Clinical requests from participants were, upon their approval, channelled to the gatekeeper for follow‐up. The PhD student role was emphasised, aiming to reduce the distance between H. J. M. and participants, where participants were perceived as experts in sharing their experiences.

## Supporting information

Supporting information.

## Data Availability

Data are not available due to ethical restrictions.
