# Supplementary material for: Participation in healthcare consultations: A qualitative study from the perspectives of persons diagnosed with hand osteoarthritis
Source: Health Expect. 2023 Mar 14;26(3):1276–86. doi: 10.1111/hex.13744 (PMC10154812; doi:10.1111/hex.13744)
Supplement: Supplementary file 1 — Supporting information. [file HEX-26--s001.pdf]

Interview guide

Symptom manifestation, initial contact with healthcare services, and diagnosis

- Examples of questions:
  - How did you notice the symptoms in your hands?
  - What did you do about it?
  - What were your reasons for contacting healthcare services?
  - What happened when you consulted healthcare services?
  - How were you informed about having hand OA?
  - What was important for you in seeking healthcare for your hand OA?

In consultations

- Examples of questions:
  - How would you describe the consultations you had with rheumatologists/occupational therapists/general practitioners?
  - Can you describe what happened during consultations?
  - How did the consultations match your expectations?
  - In what ways were you listened to regarding your hand OA condition?

Intervention and follow up

- Examples of questions:
  - What healthcare services where you provided?
  - What were your experiences with those healthcare services?
  - How did you find the recommendations that were provided to you?
  - How did you follow up on recommendations?
  - How did you find the usefulness of the interventions?
